# Biosafety and biosecurity requirements for *Orientia* spp. diagnosis and research: recommendations for risk-based biocontainment, work practices and the case for reclassification to risk group 2

**DOI:** 10.1186/s12879-019-4653-4

**Published:** 2019-12-10

**Authors:** Stuart D. Blacksell, Matthew T. Robinson, Paul N. Newton, Soiratchaneekorn Ruanchaimun, Jeanne Salje, Tri Wangrangsimakul, Matthew D. Wegner, Mohammad Yazid Abdad, Allan M. Bennett, Allen L. Richards, John Stenos, Nicholas P. J. Day

**Affiliations:** 1 Mahidol-Oxford Tropical Medicine Research Unit, Faculty of Tropical Medicine, Mahidol University, Bangkok, 10400 Thailand; 2 Centre for Tropical Medicine and Global Health, Nuffield Department of Medicine, Churchill Hospital, Oxford, OX3 7FZ UK; 3 Lao-Oxford-Mahosot Hospital-Wellcome Trust Research Unit (LOMWRU), Mahosot Hospital, Vientiane, Lao People’s Democratic Republic; 4 Armed Forces Research Institute of Medical Sciences (AFRIMS), Bangkok, 10400 Thailand; 5 Infectious Disease Research Laboratory, National Centre for Infectious Diseases, Singapore, Singapore; 6 Public Health England, Biosafety, Air and Water Microbiology Group, Porton, Salisbury, Wiltshire SP4 0JG UK; 7 Department of Preventive Medicine and Biostatistics, Uniformed Services University of the Health Sciences, Bethesda, MD 20814 USA; 8 Australian Rickettsial Reference Laboratory, Geelong Hospital, Geelong, Victoria 3220 Australia

**Keywords:** *Orientia*, Scrub typhus, Biosafety, Biocontainment, Risk group

## Abstract

Scrub typhus is an important arthropod-borne disease causing significant acute febrile illness by infection with *Orientia* spp*.*

Using a risk-based approach, this review examines current practice, the evidence base and regulatory requirements regarding matters of biosafety and biosecurity, and presents the case for reclassification from Risk Group 3 to Risk Group 2 along with recommendations for safe working practices of risk-based activities during the manipulation of *Orientia* spp. in the laboratory.

We recommend to reclassify *Orientia* spp. to Risk Group 2 based on the classification for RG2 pathogens as being moderate individual risk, low community risk. We recommend that low risk activities, can be performed within a biological safety cabinet located in a Biosafety Level (BSL) 2 core laboratory using standard personal protective equipment. But when the risk assessment indicates, such as high concentration and volume, or aerosol generation, then a higher biocontainment level is warranted. For, the majority of animal activities involving *Orientia* spp., Animal BSL 2 (ABSL2) is recommended however where high risk activities are performed including necropsies, Animal BSL (ABSL3) is recommended.

## Background

Scrub typhus is an important arthropod-borne disease causing significant acute febrile illness in the Asia-Pacific region [1] caused by *Orientia tsutsugamushi.* Other members of scrub typhus group orientiae [2] include *Candidatus* Orientia chuto from United Arab Emirates [3] and Kenya [4], and the incompletely characterized orientiae that cause scrub typhus in Chile [5–7]. *Orientia* spp. are members of the family Rickettsiaceae in the order Rickettsiales, are Gram-negative, obligate, intracellular bacteria and are transmitted by arthropod vectors [8, 9].

The laboratory diagnosis of scrub typhus requires the detection of antibodies against and/or nucleic acids of *O. tsutsugamushi* using serological techniques and PCR, respectively [9, 10]. In vitro culture of *Orientia* spp. is generally not required for diagnostic purposes, though culture is required to provide the raw materials for diagnostic assays, whole genome sequencing, and the study of growth characteristics. The in vitro or in vivo culture of *Orientia* spp. has the potential hazard of laboratory-acquired infections (LAIs) of staff by means of parenteral inoculation such as accidental self-inoculation, cut, animal bite or scratch, or exposure to aerosols from laboratory procedures or accidents [11–13]. Furthermore, *Orientia* spp. are sensitive to antibiotics including doxycycline and azithromycin [14, 15].

Using a risk-based approach, this review examines the evidence base, regulatory requirements and current practice, regarding biosafety and biosecurity practises, and argues for the reclassification from Risk Group 3 to Risk Group 2 along with recommendations for safe practices of risk-based activities during the manipulation of *Orientia* spp. in the laboratory.

### Risk group classification and biocontainment regulations for *Orientia* spp. research

Risk Groups (RG) (known as Hazard Groups (HG) in the United Kingdom) are used as biosafety “shorthand” to quantify the degree of hazard associated with a pathogen or toxin. Although there is no universal RG classification there is general agreement amongst countries on RG principles (Table 1). Briefly, RG1 pathogens are those of low individual and community risk. RG2 pathogens represent the largest group and have the characteristics of moderate individual risk and low community risk, and there is general agreement that they are classified as those that “*can cause human disease and might be a hazard to workers and effective treatment and preventive measures are available (antibiotics or vaccine) and the risk of spread of diseases in the community caused by these pathogens is low”*. RG3 pathogens are considered high individual risk with limited/moderate community risk and “*likely to cause serious disease and may present a risk of spreading to the community, but there is usually effective prophylaxis or treatment available*” (Table 1).

**Table 1:** Examples of worldwide biological Risk Group classifications

Country or region	Risk Group 1	Risk Group 2	Risk Group 3	Risk Group 4
Australian/New Zealand Standard [16]	Low individual and community risk A microorganism that is unlikely to cause human or animal disease.	Moderate individual risk, limited community risk A microorganism that is unlikely to be a significant risk to laboratory workers, the community, livestock, or the environment; laboratory exposures may cause infections, Effective treatment and preventive measures are available, and the risk of spread is limited.	High individual risk, limited to moderate community risk A microorganism that usually causes serious human or animal disease and may present a significant risk to laboratory workers. It could present a limited to moderate risk if spread in the community or the environment, but there are usually effective preventive measures or treatment available.	High individual and community risk A microorganism that usually produces life-threatening human or animal disease, represents a significant risk to laboratory workers and may be readily transmissible from one individual to another. Effective treatment and preventive measures are not usually available.
Belgium [17]	Micro-organisms known as nonpathogenic for the man, the animal, the plant and not-harmful for the environment or presenting a negligible risk for the man and the environment at the laboratory scale. This class includes, beside organisms whose harmlessness was proven, strains which can be allergens and opportunistic pathogens	Micro -organisms that can cause human disease and might be a hazard for directly exposed persons; they are unlikely to spread to the community. There is usually effective prophylaxis or treatment available.	Micro -organisms that can cause severe human disease and present a serious hazard for directly exposed persons. They may present a risk of spreading to the community. There is usually effective prophylaxis or treatment available.	Micro -organisms that cause severe human disease and are a serious hazard for directly exposed persons. They may present a high risk of spreading to the community. There is usually no effective prophylaxis or treatment available.
Canada [18]	Low individual and community risk A microorganism, nucleic acid, or protein that is either a) not capable of causing human or animal disease; or b) capable of causing human or animal disease, but unlikely to do so. Those capable of causing disease are considered pathogens that pose a low risk to the health of individuals or animals, and a low risk to public health and the animal population.	Moderate individual risk, low community risk A pathogen or toxin that poses a moderate risk to the health of individuals or animals, and a low risk to public health and the animal population. These pathogens are able to cause serious disease in a human or animal but are unlikely to do so. Effective treatment and preventive measures are available and the risk of spread of diseases caused by these pathogens is low.	High individual risk, low community risk A pathogen that poses a high risk to the health of individuals or animals, and a low risk to public health. These pathogens are likely to cause serious disease in a human or animal. Effective treatment and preventive measures are usually available and the risk of spread of disease caused by these pathogens is low for the public. The risk of spread to the animal population, however, can range from low to high depending on the pathogen.	High individual risk, high community risk A pathogen that poses a high risk to the health of individuals or animals and a high risk to public health. These pathogens are likely to cause serious disease in a human or animal, which can often lead to death. Effective treatment and preventive measures are not usually available and the risk of spread of disease caused by these pathogens is high for the public. The risk of spread of disease to the animal population, however, ranges from low to high, depending on the pathogen.
European Economic Community Directive 2000/54/EC and Directive 90/679/EEC [19]	Biological agent means one that is unlikely to cause human disease.	Biological agent means one that can cause human disease and might be a hazard to workers; it is unlikely to spread to the community; there is usually effective prophylaxis or treatment available.	Biological agent means one that can cause severe human disease and present a serious hazard to workers; it may present a risk of spreading to the community, but there is usually effective prophylaxis or treatment available.	Biological agent means one that causes severe human disease and is a serious hazard to workers; it may present a high risk of spreading to the community; there is usually no effective prophylaxis or treatment available.
Germany [19]	Biomaterials, where it is unlikely that they cause disease in humans	Biomaterials that can cause human disease and might be a hazard to workers; Dispersal in the population is unlikely; Effective prophylaxis or treatment is normally possible	Biomaterials, which cause severe human disease and present a serious hazard to workers; The risk of spread to the community, but there is usually effective prophylaxis or treatment available	Biological agents which cause severe human disease and are a serious hazard to workers; The risk of spread in the population is high under certain circumstances; there is usually no effective prophylaxis or treatment.
United Kingdom [20]	Unlikely to cause human disease	Can cause human disease and may be a hazard to employees; It is unlikely to spread to the community and there is usually effective prophylaxis or treatment available.	Can cause severe human disease and may be a serious hazard to employees; It may spread to the community, but there is usually effective prophylaxis or treatment available.	Causes severe human disease and is a serious hazard to employees; It is likely to spread to the community and there is usually no effective prophylaxis or treatment available

At present, all members of *Orientia* spp*.* and the related *Rickettsia* spp*.* are classified as RG3 pathogens or equivalent in the United States, United Kingdom, Australia, Singapore, Germany, Switzerland, Belgium, and the European Union [17, 19]. As such, significant biosafety controls have been placed on their manipulation, propagation and storage. Interestingly, in many regions where the disease is endemic, there are few statutory regulations or guidelines regarding biosafety, as well as a lack of clear guidance on the level of biocontainment required for laboratory work on *Orientia* spp. In the absence of clear local regulations, government regulatory authorities often look to the USA or UK regulations, or elsewhere, to provide the basis for biosafety and biocontainment. There is often an imperative from donors to meet specific biosafety and biocontainment guidelines prior to the disbursement of funds. The following is a short description of the guidelines and regulations as specified by national regulators and a summary is provided in Table 2.

**Table 2:** Biocontainment and mitigation regulations for *Orientia* spp.

			Laboratory studies	
Country	Regulations	Activity	Low-risk	Enhanced-risk
USA	BMBL5th Ed. [21]	Clinical	“BSL2 practices, biocontainment equipment, and facilities are recommended for non-propagative laboratory procedures, including serological and fluorescent antibody procedures, and for the staining of impression smears.	New species are being described frequently and should be evaluated for appropriate biocontainment on a case-by-case basis. Because of the proven value of antibiotic therapy in the early stages of rickettsial infection, it is essential that laboratories have an effective system for reporting febrile illnesses in laboratory personnel, medical evaluation of potential cases and, when indicated, institution of appropriate antibiotic therapy.
		Research	*R prowazekii; R typhi (R. mooseri); Orientia (Rickettsia) tsutsugamushi and Spotted Fever Group agents of human disease; R rickettsii, R conorii, R akari, R australis, R siberica, and R japonicum -* BSL3 practices, biocontainment equipment, and facilities are recommended for all other manipulations of known or potentially infectious materials, including necropsy of experimentally infected animals and trituration of their tissues, and inoculation, incubation, and harvesting of embryonated eggs or cell cultures. *R. montana, R. rhipicephali, R. belli*, and *R. canada,-* are not known to cause human disease and may be handled under BSL2 conditions.	
UK	ACDP [20]	Clinical	“Microbiology laboratories offering a diagnostic service to a hospital will from time to time isolate a hazard group 3 pathogen when working at CL2. Once identified, work on such isolates and on material known or suspected to contain hazard group 3 biological agents must be conducted in a CL 3 laboratory, unless the agent is specifically identified as an exemption in the ACDP Second supplement 2000.”	“It is recognised that pathogens may be present in specimens which, had they been identified, would need to be handled at a higher level of containment. If such pathogens are identified during the course of work at CL 2, all further work on the specimen or associated specimens must be conducted at a higher biocontainment level, usually CL 3 or exceptionally CL 4. If higher biocontainment level facilities are not available, the isolate should be sent to an appropriate laboratory, or be destroyed. If it is suspected, for example from a clinical history, that a specimen may contain hazard group 3 biological agents, all work on that specimen or other specimens from that patient must be conducted at CL3.”
		Research		*Rickettsia spp* (specified as the following *R. akari, R canada, R conorii, R montana, R prowazekii*, *R rickettsii, R (Orientia) tsutsugamushi, R sennetsu, R typhi*) are classified as a Hazard Group 3 Pathogen “the minimum biocontainment level… shall be…— level 3 for activities which involve working with a Group 3 biological agent” and so in the UK all work with known *Orientia* spp. must be conducted in a CL3/BSL 3 laboratory.
Australia	AS/NZS 2243.3.2010 [16]	Clinical	Clinical specimens are generally regarded as low risk and handled under BSL2 conditions. Very few laboratories attempt cell culture isolation of *Orientia* spp. from clinical specimens and the recovery rate of viable organisms is very low. However, once an isolate has been identified the specimen must be subsequently be handled at BSL3.	There may be a need for further characterisation of a clinical isolate with respect to typing and as such these activities need to be performed at BSL3.
		Research	Inactivated *Orientia* isolates can be handled at BSL2	All viable Orientia/rickettsial agents need to be handled at BSL3.
Singapore	BATA 2005 [22]	Clinical	For samples where the diagnosis is unknown, diagnostic testing that does not result in amplification of the pathogen tested for can be performed in a BSL2 environment. Laboratories conducting clinical diagnostics of *Rickettsia* and *Orientia* spp. are required to conduct risk assessment and provide appropriate measures and safeguards to significantly reduce risk of infection of laboratory staff.	In enhanced risk situation, the work environment will need to be BSL3. This covers all work that involves “live” organisms in the sample or culture, whereby no prior approved steps have been applied to the material that would render the pathogen inert and non-infectious.
		Research	For research work on live samples and cultures, the work environment is required to be at BSL3. Large scale culture (> 10 l) is prohibited without written authorization of the Director of Medical Services, Ministry of Health Singapore.	
Thailand	Pathogens and Animals Toxins Act 2015 [23]	Clinical	Routine diagnostic laboratory processing within hospital laboratories can be performed in BSL-2 laboratories with strict adherence to Good Laboratory Practice guidelines.	Culturing with the aim of producing large amounts of bacteria should be performed in BSL3 laboratories or equivalent safety level (BSL2 enhanced).
		Research	As per clinical activity	

*BSL* biological safety level, *ACDP* advisory committee on dangerous pathogens, *CL* biocontainmentlevel, *AS/NZS* Australian/New Zealand Standards, *BATA* biologicals and toxins act

Physical biocontainment is generally classified as biosafety levels (BSL1–4) although there are differing national standards and regulations. To increase standardisation, the World Health Organization (WHO) has recently revised the WHO Laboratory Biosafety Manual (LBM 4th edition) using a risk-based approach [24] with three levels of biocontainment; 1) Core, 2) Heightened Control Measures (HCM) and 3) Maximum containment.

#### USA regulations

*Orientia tsutsugamushi* and *Rickettsia* spp. (e.g. *R. prowazekii*, *R. typhi*, *R. rickettsii*, *Rickettsia conorii*, *R. akari*, *R. australis*, *R. sibirica*, and *R. japonica*) are classified as RG3 organisms and are detailed in the BMBL 5th edition [21] in regards to their control as follows (p197–198). “BSL2 practices, biocontainment equipment, and facilities are recommended for non-propagative laboratory procedures, including serological and fluorescent antibody procedures, and for the staining of impression smears. BSL3 practices, biocontainment equipment, and facilities are recommended for all other manipulations of known or potentially infectious materials, including necropsy of experimentally infected animals and trituration of their tissues, and inoculation, incubation, and harvesting of embryonated eggs or cell cultures. Animal BSL (ABSL) 2 practices, biocontainment equipment, and facilities are recommended for the holding of experimentally infected mammals other than arthropods. ABSL3 practices, biocontainment equipment, and facilities are recommended for animal studies with arthropods naturally or experimentally infected with rickettsial agents of human disease. Several species, including *R. montanensis, R. rhipicephali*, *R. bellii,* and *R. canadensis*, are not known to cause human disease and may be handled under BSL2 conditions.”

#### UK regulations

In the UK, the Advisory Committee on Dangerous Pathogens (ACDP) advises the Health and Safety Executive (HSE) on aspects of hazards and risks to workers and others from pathogen exposure pathogens. All *Rickettsia* spp. (*R. akari*, *R. canadensis*, *R. conorii*, *R. montanensis*, *R. prowazekii*, *R. rickettsii*, *R.* (*Orientia*) *tsutsugamushi*, *R. sennetsu*, *R. typhi*) are classified as HG 3 pathogens in the Approved List of Biological agents 2013 (ACDP/HSE) [20]. The Control of Substances Hazardous to Health Regulations 2002 (Schedule 3, Part I, Paragraph 3 (4) (b)) state that “the minimum biocontainment level… shall be… level 3 for activities which involve working with a Group 3 biological agent” and so, in the UK, all work with confirmed *Orientia* spp. must be conducted in a Biocontainment Level (CL = UK BSL equivalent) 3 laboratory. The position is less straightforward when specimens from suspected cases of *Orientia* spp. infection are handled in a clinical laboratory for diagnostic purposes (Table 2).

#### Australia

In Australia, laboratories are governed by the Australian/New Zealand Standards AS/NZS which outline the safety principles in laboratories, AS/NZS 2243.3.2010 Safety in Laboratories Part 3: Microbiological Safety and Biocontainment [16]. Clinical specimens with unconfirmed microbial aetiology are regarded as low risk and handled at BSL2. However, once a high-risk pathogen has been isolated from a specimen, it must then be handled at a higher biocontainment level. Although rickettsiae are mentioned as RG3 pathogens there is no mention of *Orientia* spp. in the Standard. However, *Orientia* spp. were historically labelled as a *Rickettsia* spp. and this perhaps indicates the need for this standard to be updated.

#### Singapore

The legislation that governs the possession, use, import, transhipment, transfer, and transportation of biological agents in Singapore falls under the Biologicals and Toxins Act (BATA), that was originally enacted in 2005 [22]. Rickettsial organisms of the genus *Rickettsia* are classified within the act as First Schedule biological agent meaning “Risk Group 3 Biological Agents which can cause serious disease which is of high risk to the individual”. Members of the genus *Orientia* are not specifically mentioned but they are generally viewed as similar to *Rickettsia* spp*.* and thus require the same restrictions. Although the legislation does not specify the restrictions of the environment where a biological agent should be worked on, organisms are organised into “Schedules” that share similar restrictions (i.e., First Schedule agents may require BSL3, Second Schedule agents may require BSL4, Third and Fourth Schedule may require BSL2). This allows for the testing of normally accepted RG3 pathogens such as *Rickettsia* and *Orientia* spp*.* from non-amplified diagnostic samples to potentially be performed in a BSL2 environment should the appropriate approval be granted after a review of the planned work environment and the conduct of appropriate risk assessments.

#### Thailand

The Royal Thai Government enacted the Pathogens and Animal Toxins Act in 1982 that is administered by the Ministry of Public Health and focuses on the regulation and security of pathogens and toxins [23]. It has subsequently been updated in 2001 and 2015 with the latter superseding the previous iterations. Pathogens are designated into 4 risk groups, numbered 1 to 4, corresponding to low, moderate, high, and very high risk, respectively. These pathogens were further defined in the official Announcement of the Ministry of Public Health “List of controlled pathogens under section 18 (of the Act)” 2017 [25]. Group 2 pathogens (moderate risk) can be handled under BSL2 conditions and include *R. africae*, *R. bellii*, *R. canadensis*, *R. felis*, *R. helvetica*, *R. honei*, *R. japonica*, *R. montanensis*, *R. parkeri*, *R. quintana*, *R. rhipicephali*, *R. sennetsu* and *R. slovaca*. Group 3 pathogens include *R. akari*, *R. australis*, *R. conorii*, *R. prowazekii*, *R. rickettsii*, *R. sibirica*, *R.* (*Orientia*) *tsutsugamushi,* and *R. typhi*. Routine diagnostic laboratory processing within hospital laboratories can be performed in BSL-2 laboratories with strict adherence to Good Laboratory Practice guidelines. Culturing with the aim of producing large amounts of bacteria should be performed in BSL3 laboratories or equivalent safety level (BSL2 enhanced).

#### Other countries and regions

Germany, Switzerland, Belgium [17] and the European Union classify *Orientia tsutsugamushi* as an RG3 pathogen [19]. Canada does not provide a RG classification in its Pathogen Safety Data Sheet resource [18].

### Risk-assessment for *Orientia* spp.

*The case for reclassification of Orientia spp. to RG2 using a risk-based justification.*

Using the broad classification for RG2 pathogens of moderate individual risk, low community risk that “*can cause human disease and might be a hazard to workers and effective treatment and preventive measures are available (antibiotics or vaccine) and the risk of spread of diseases in the community caused by these pathogens is low”*, a strong argument can be made to reclassify *Orientia* spp. to RG2. Certainly, *Orientia* spp. do not fit the characteristics of RG3 pathogens which are considered high individual risk, limited/moderate community risk with the key difference being the risk of spread of the disease in the community. We therefore recommend reclassification of *Orientia* spp. to RG2 by all countries.

#### Recommended risk assessments for Orientia spp. research activities

Risk associated with *Orientia* spp. individual research activities and laboratory procedures.

Laboratory activities associated with risk of accidental exposure of personnel and the environment to *Orientia* spp. include (1) the processing of clinical specimens from scrub typhus patients and, (2) in vitro culture of *Orientia* spp. in mammalian cell cultures or embryonated chicken eggs and associated downstream manipulations including centrifugation, harvesting of cells and supernatants and, carrying cultures to and from incubators to a Biological Safety Cabinet (BSC) [11–13].

LOW risk activities for both laboratory staff and environmental contamination in case of an accident would be small-scale processing of suspected scrub typhus patient samples such as initial in vitro inoculation or subsequent passage of patient samples on to cell cultures with a guideline of a total of ≤100 ml (in a single or multiple culture vessels) at a concentration of ≤10^6^ cfu/ml *Orientia* spp. organisms. This may also include small-scale passaging of reference cultures.

MEDIUM risk activities may include those such as medium-scale passaging of reference cultures or medium-scale passage of clinical *Orientia* spp. isolates to increase the viable organism concentration thereby increasing the risk to the laboratory staff whilst maintaining a low risk of escape to the environment in case of an accident. Suggested guidelines for medium risk activities would be incubation and manipulation guideline of a total of ≤500 ml (in a single or multiple culture vessels) at a concentration of ≤10^6^ cfu/ml *Orientia* spp. organisms.

HIGH risk activities involve increased volume and concentration of the *Orientia* spp. organisms manipulated such as in the case of reagent production which may involve large-scale production of highly concentrated *Orientia* spp. as well as manipulation with increased inherent risk such centrifugation and concentration techniques, as there is an increased risk to the laboratory staff and of escape to the environment in case of an accident. Suggested guidelines for high risk activities would be incubation and manipulation guideline of a total of > 500 ml (in a single or multiple culture vessels) at a concentration of > 10^6^ cfu/ml *Orientia* spp. organisms.

The determination of the risk level should be made by a biosafety professional in conjunction with stakeholders including principal investigator and laboratory management as the risk mitigation measures are likely to have significant infrastructure and financial implications.

Risk associated with *Orientia* spp. during experimental animal activities

Experimental infection of animals (vertebrates and invertebrates) with *Orientia* spp. presents a different range of infection risks than those of laboratory activities. Vertebrates used for experimental infections are predominantly rodents [26, 27], guinea pigs [28] and non-human primates [29, 30] while chiggers (*Leptotrombidium* spp.) [29] have been the dominant genus of invertebrates. The risks may be broadly characterised as aerosol and parenteral exposures. Aerosol exposures would normally be associated with necropsy activities, cage and bedding changing (if agents is secreted in urine and faeces), and infectious waste exposures [31]. Parenteral exposures would normally be due to bites, cuts, and scratches from animals or sharp equipment [11, 13, 31]. All such activities if performed on a small-scale would normally be considered MEDIUM risk. However, if the number of animals significantly increase or larger animal species are used, then risk would increase to HIGH. As mentioned above, in the case of increased risk activities, this determination should be made by a biosafety professional in conjunction with stakeholders. For MEDIUM risk procedures where there is minimal aerosol generation then Animal Biosafety Level 2 (ABSL2) is recommended however where HIGH risk activities are performed including necropsies, Animal Biosafety Level 3 (ABSL3) is recommended. Mitigation strategies for medium- and high-risk animal activities based on the US BMBL 5th edition regulations [21] are presented in Table 3.

**Table 3:** Mitigation strategies for medium- and high-risk animal activities based on the US BMBL 5th edition regulations [21]

Mitigation strategy	Experimentally-infected animals	Arthropods and necropsy of infected animals
Personnel protection	Experimental *Orientia* spp. and any work on isolates of the organism would be undertaken in a Class II BSC^a^ meaning that the risk of airborne exposure of staff is minimal. If a Class II BSC is not available and there is a risk of aerosol exposure, staff should be fitted with and wear an N-95 respirator. All work on samples that are suspected of containing *Orientia* spp. and any work on isolates of the organism would be undertaken by staff wearing gloves with avoidance of sharps wherever possible, minimising the risk of inoculation. Outer garments, such as lab coats or Tyvek overalls, should be worn during work which has the potential to produce splashes or aerosols of bacteria. Workers can be provided with a medical alert card to take to medical clinics should an accidental exposure occur. This card contains information about the nature of the work, the agent being worked with, and appropriate medical management to help inform and educate health care providers who may not be familiar with scrub typhus or laboratory animals.	As for experimentally-infected animals
Primary containment	Class II BSC	As for experimentally-infected animals
Secondary biocontainment	ABSL2	BSL3/ABSL3
Animal Handling	Appropriate PPE for the species of animal should be worn. Working with non-human primates (NHP) poses a risk of infection with Macacine herpesvirus and PPE should be utilized to prevent infection with this virus. If using rodents, the risk of bites can be mitigated by acclimatizing the animals to handling or by wearing bite-resistant gloves. Non-human primates should also be trained and acclimatized to the various procedures encountered in the course of the protocol. Use of pole, collar, and chair restraint devices minimize direct animal handling for examination, injections/inoculations, and treatments. If this is not possible, NHP should be anesthetized using an appropriate anaesthetic protocol for the procedure. Necropsy of scrub typhus-infected animals should be undertaken in appropriate facilities based on the infection status of the animal. If the animal is infectious, (i.e. active bacteraemia or infection), the animal should be necropsied in a Class II BSC and/or within an ABSL3 or BSL3 laboratory. If the animal is non-infectious, standard necropsy procedures can be followed.	As for experimentally-infected animals
Disinfection (any of the following options)	Autoclave – 121 °C for 30 min Dry heat - 160-170 °C for at least 60 min Chemical (At least 30 min contact time) 1% Virkon (surfaces) or 2% Virkon (liquids) 1% sodium hypochlorite 70% ethanol, Glutaraldehyde Formaldehyde Quaternary ammonium disinfectants.	As for experimentally-infected animals
Training	Additional, specialized training should occur when working with laboratory animals. The type of training may vary depending on the regulatory requirements of the country in which the work is being performed. Staff working with NHP should receive training about Macacine herpesvirus since this is an extremely dangerous zoonotic disease that can lead to death if not treated. Staff should be trained and proficient in animal handling, restraint, sample collection, treatment administration, necropsy procedures, and other experimental procedures on animals before working with scrub typhus-infected animals.	As for experimentally-infected animals

^a^*BSC* biological safety cabinet

#### Recommendations for risk-based biocontainment levels for Orientia spp. laboratory activities

Risk-based assessment of laboratory activities for *Orientia* spp., when classified as a RG2 pathogen, do not support the need for BSL3 biocontainment for LOW-risk routine, low-volume culturing of *Orientia* spp. and downstream activities and most MEDIUM-risk activities. Therefore, these activities can be performed at BSL2 biocontainment level in a BSC with standard Personal Protective Equipment (PPE) which would be consistent with WHO HCM. However, for HIGH-risk activities where high concentrations and large volumes of live organisms are involved such as in the case of reagent production, the risk assessment may suggest performing the activities at a higher level of secondary containment, such as BSL3 containment, and PPE may need to include respirators if primary biocontainment equipment (e.g. BSC) are not available or easily able to be used. Stocks of reference cultures at high concentrations require sufficient levels of physical biosecurity including locked freezers, stored behind locked doors with restricted/vetted personnel access to prevent the theft of pure cultures that may be used for nefarious purposes. Risk-based recommendations for biocontainment and associated mitigation strategies for low- and high-risk *Orientia* spp. laboratory activities are presented in Table 4.

**Table 4:** Risk-based recommendations for biocontainment and associated mitigation strategies for low- and high-risk laboratory activities

Mitigation strategy	Low^a^ to Medium^b^-risk laboratory activities	High-risk laboratory activities ^c^
Personnel protection	Gown Gloves Eye protection Covered shoes Avoidance of sharps	Respiratory protection Gown Gloves Eye protection Covered shoes Avoidance of sharps
Primary containment	Class II BSC (annually certified) Aerosol generating procedures (i.e., centrifugation) devices with bioseal (gaskets buckets and corresponding lids (screw or clip type). Buckets may only be opened in the BSC	Class II BSC (annually certified) Aerosol generating procedures (i.e., centrifugation) devices with bioseal (gaskets buckets and corresponding lids (screw or clip type). Buckets may only be opened in the BSC
Secondary biocontainment	Biosafety Level 2 or Core	Biosafety Level 3 or Heightened Control Measures
Disinfection (any of the following options)	Autoclave – 121 °C for 30 min Dry heat - 160-170 °C for at least 60 min Chemical (at least 30 min contact time) 1% Virkon (surfaces) or 2% Virkon (liquids) 1% sodium hypochlorite 70% ethanol Glutaraldehyde Formaldehyde Quaternary ammonium disinfectants	Autoclave – 121 °C for 30 min Dry heat - 160-170 °C for at least 60 min Chemical (at least 30 min contact time) 1% Virkon (surfaces) or 2% Virkon (liquids) 1% sodium hypochlorite 70% ethanol Glutaraldehyde Formaldehyde Quaternary ammonium disinfectants
Training	Staff need to be trained and demonstrate proficiency in GMPP	Staff need to be trained and demonstrate proficiency in GMPP

*BSC* biological safety cabinet, *GMPP* good microbiological practises and procedures

^a^Low-risk – ≤100 ml (in a single or multiple culture vessels) at a concentration of ≤10^6^ cfu/ml *Orientia spp*. organisms

^b^Medium-risk - ≤500 ml (in a single or multiple culture vessels) at a concentration of ≤10^6^ cfu/ml *Orientia spp*. organisms

^c^High-risk - > 500 ml (in a single or multiple culture vessels) at a concentration of > 10^6^ cfu/ml *Orientia spp*. organisms

## Discussion

Until now, *Orientia* spp*.* and *Rickettsia* spp*.* have been classified as RG3 pathogens resulting in substantial controls placed on their growth, handling and storage. This RG3 classification may have evolved from association with the more pathogenic *R. rickettsii* and *R. prowazekii* –which were classified by the United States as Biological Select Agents in 2007 due to their biothreat status [21, 32], however, only *R. prowazekii* retains this status in the current Select Agents list [33]. *Orientia* spp*.* are classified as RG3 pathogens by the USA, European Union, Australia, New Zealand, Belgium, Germany, Switzerland and Thailand [19, 25]. While *Orientia* spp*.* and many *Rickettsia* spp. have the potential to cause serious infections, it is difficult to justify their status as RG3 pathogens because they do not spread by person-to-person contact and are amenable to antibiotic treatment.

Further confusion around biosafety requirements for *Orientia* spp*.* laboratory investigations is the fallacy that RG status is equivalent to biocontainment level [24]. This misinterpretation has resulted in the incorrect assumption that BSL3 biocontainment is a mandated requirement for all *Orientia* spp*.* manipulations, without considering the procedural risks. BSL3 biocontainment laboratories fundamentally differ from BSL2 biocontainment as they provide directional airflow via a negative pressure gradient and HEPA filtered exhaust air that provides protection to the outside environment from aerosol contamination in the event of a catastrophic laboratory accident. A consequence of the need for complex engineering solutions to provide adequate BSL3 biocontainment is that such laboratories are expensive to build, maintain, and manage. Furthermore, such laboratories typically have high electricity costs to maintain heating, ventilation and air-conditioning (HVAC) requirements which further compounded by the regulatory necessity for single pass non-recirculated air. Building and operating a BSL3 laboratory places financial strain on already stretched budgets, and those wishing to maintain regulatory compliance often choose not perform such activities at a lower biocontainment levels for fear of not complying with national laws, funder requirements, or incorrectly perceiving the activities to be too “high risk” to be performed.

It is likely that the requirement for BSL3 biocontainment has had unintended consequence of impeding scrub typhus research and vaccine development [34]. Laboratory and regulatory administrators have taken an overly cautious attitude to the regulatory aspects of research on *Orientia* spp. and other rickettsial pathogens. This has resulted in the proliferation of high-biocontainment laboratories in both relatively-wealthy and developing countries. The need for high-biocontainment laboratories for *Orientia* spp*.* propagation has been overstated and the mistaken requirement to perform all such work in BSL3 laboratory facilities through rules imposed by national governments, or foreign institutions providing donor funds has further compounded the issue.

The evidence presented here demonstrates that work with *Orientia* spp*.* and *Rickettsia* spp. requires a risk-based consideration when selecting biosafety and biocontainment controls. Using a blanket RG3/BSL3 approach is inappropriate and restrictive except when the risks are clearly high and the controls justified. The WHO LBM 4th edition [24], uses a risk-based approach and stipulates that only high-risk activities be performed in HCM laboratories, which may include (but is not restricted to) BSL3 laboratory facilities.

The evidence from scrub typhus LAI reports [13, 31], clearly supports the proposal that low risk activities, can be safely performed within a BSC located in BSL2 core laboratory (i.e., HCM laboratory). When warranted, the requirement for increased secondary containment, PPE and enhanced practices should be maintained. This is supported by the fact that scrub typhus LAIs have not been reported for nearly 20 years [13], and exposure of laboratory staff to infectious materials and their products are ameliorated by rigorous biological safety mitigation strategies through administrative controls including a focus on good microbiological practises and procedures, competency assessment, was well as judicious selection of PPE.

## Conclusions

We recommend the reclassification of *Orientia* spp. to RG2 based on the classification of RG2 pathogens as being moderate individual risk and low community risk. Furthermore, using a risk-based approach, we recommend that low risk activities can be performed within a BSC located in a BSL2 core laboratory (i.e., HCM laboratory) and high-risk activities, such as those involving aerosol generation or high bioburden of bacteria, would require the use of BSL3 laboratory facilities. The majority of animal activities involving *Orientia* spp. would still require ABSL3 containment. Consideration should also be given to the risk-group reclassification of selected *Rickettsia* spp. using the same risk-based approach.

## Data Availability

All data generated or analysed during this study are included in this published article.

## Abbreviations

ABSL: Animal Biological Safety Level
BSC: Biological Safety Cabinet
BSL: Biological Safety Level
GMPP: Good Microbiological Practises and Procedures.
HCM: Heightened Control Measures
LAI: Laboratory-acquired infection
LBM: Laboratory Biosafety Manual
PPE: Personal Protective equipment
RG: Risk Group
WHO: World Health Organization
